# Small Interfering RNA Targeted to ASPP2 Promotes Progression of Experimental Proliferative Vitreoretinopathy

**DOI:** 10.1155/2016/7920631

**Published:** 2016-06-09

**Authors:** Xiao-Li Chen, Yu-Jing Bai, Qin-Rui Hu, Shan-Shan Li, Lv-Zhen Huang, Xiao-Xin Li

**Affiliations:** ^1^Ophthalmology Department, Peking University People's Hospital, Key Laboratory of Vision Loss and Restoration, Ministry of Education, Beijing Key Laboratory for the Diagnosis and Treatment of Retinal and Choroid Diseases, Beijing 100044, China; ^2^Department of Ophthalmology, QiLu Hospital, Shandong University, Jinan, Shandong 250012, China

## Abstract

*Background*. Epithelial-mesenchymal transition (EMT) of retinal pigment epithelium (RPE) is vital in proliferative vitreoretinopathy (PVR) development. Apoptosis-stimulating proteins of p53 (ASPP2) have recently been reported to participate in EMT. However, the role of ASPP2 in PVR pathogenesis has not been identified.* Methods*. Immunohistochemistry was used to investigate the expression of ASPP2 in epiretinal membranes of PVR patients. ARPE-19 cells were transfected with ASPP2-siRNA, followed with measurement of cell cytotoxicity, proliferation, and migration ability. EMT markers and related inflammatory and fibrosis cytokines were measured by western blot or flow cytometry. Additionally, PVR rat models were induced by intravitreal injection of ARPE-19 cells transfected with ASPP2-siRNA and evaluated accordingly.* Results*. Immunofluorescence analysis revealed less intense expression of ASPP2 in PVR membranes. ASPP2 knockdown facilitated the proliferation and migration of RPE cells and enhanced the expression of mesenchymal markers such as alpha smooth muscle actin, fibronectin, and ZEB1. Meanwhile, ASPP2-siRNA increased EMT-related and inflammatory cytokines, including TGF-*β*, CTGF, VEGF, TNF-*α*, and interleukins. PVR severities were more pronounced in the rat models with ASPP2-siRNA treatment.* Conclusions*. ASPP2 knockdown promoted EMT of ARPE-19 cells* in vitro* and exacerbated the progression of experimental PVR* in vivo*, possibly via inflammatory and fibrosis cytokines.

## 1. Introduction 

Proliferative vitreoretinopathy (PVR) remains a sophisticated obstacle that complicates vitreoretinal pathologies such as retinal detachment and posterior ocular trauma. Fibrotic epiretinal membranes are common in most PVR cases, and intraretinal changes are crucial in the severe forms [1]. Cells including retinal pigment epithelial (RPE) cells, glial cells, phagocytes, and fibroblasts have been identified in epiretinal membranes from PVR patients as well as experimental animal models [2–4]. Nowadays, PVR is considered a multifactorial disease in which both RPE cells and glial cells play a crucial role [5]. RPE cells are acknowledged to play a pivotal role during PVR through morphological and phenotypic transdifferentiation [2, 6]. RPE is a monolayer of differentiated cells located between the neural retina and Bruch's membrane, performing essential functions for the maintenance of the normal visual process [7]. However, during retinal detachment or ocular trauma, RPE cells released from the monolayer undergo epithelial-mesenchymal transition (EMT) and dedifferentiate into mesenchymal-like phenotypes. Once involved in this process, RPE cells can proliferate, migrate, and transform into myofibroblasts, a major cell type for contraction in epiretinal membranes [8]. Besides, inflammation is a major component of PVR as cytokines and factors in the vitreous could come into contact with intraretinal and RPE cells [5, 9].

EMT is the process by which epithelial cells undergo morphologic transition to mesenchymal cells such as fibroblasts and myofibroblasts, and EMT occurs during embryonic development, tumor metastasis, wound healing, and organ fibrosis [10]. This transition is characterized by the loss of cell-cell contacts, disruption of epithelial polarity, downregulation of characteristic epithelial cell markers such as E-cadherin, and upregulation of mesenchymal markers including fibronectin and alpha smooth muscle actin (*α*-SMA) [11]. Furthermore, EMT is associated with enhanced cell migration, subsequently aggravating progressive fibrotic diseases of the eye, heart, kidney, liver, and lung [12, 13]. Both* in vivo* and* in vitro* studies suggest that EMT of RPE cells has been a primary contributor to PVR [14–16], and EMT could be induced by certain inflammatory mediators [17]. However, the mechanisms underlying EMT have remained elusive.

Apoptosis-stimulating proteins of p53 (ASPP), a family of ankyrin repeat and proline-rich domain-containing proteins, consist of three members: ASPP1, ASPP2, and iASPP [18]. ASPP2, the best-characterized member of the family, was identified as a p53-binding protein in a yeast two-hybrid screen [19]. ASPP2 is known as an important tumor suppressor, p53 activator, PAR3 binding partner, and regulator of epithelial plasticity [20, 21]. Reduced ASPP2 expression in human cancers not only induces EMT to facilitate the metastasis of cancer cells, but it also helps cancer cells survive p53-mediated apoptosis [22].

Since P53 codon 72 polymorphism (rs1042522) is associated with a higher risk of PVR developing after a primary retinal detachment and p53 gene expression is involved in PVR progression [23, 24], it would be of interest to explore whether ASPP2 play a role in the development of PVR.

For now, most studies have focused on the ASPP2 function in relation to tumor biology or in neuronal apoptosis [25, 26], but the role of ASPP2 in proliferative retinal diseases has not been established.

In the present study, we investigated the effects of ASPP2 on regulating EMT of a human RPE cell line (ARPE-19) cells by transfecting ASPP2-specific short interfering (si) RNA. Meanwhile, we injected ARPE-19 cells transfected with chemically modified ASPP2-specific siRNA into rat vitreous cavities to explore its role in the pathogenesis of PVR.

## 2. Methods 

### 2.1. Cell Culture and Reagents

A human retinal pigment epithelial cell line (ARPE-19, CRL-2302; American Type Culture Collection (ATCC), Manassas, VA, US) was cultured in Dulbecco's modified Eagle's medium (DMEM)/F12 medium (HyClone; Hyclone, Grand Island, NY, US) containing 10% fetal bovine serum (FBS; Gibco, Grand Island, NY, US) under a humidified atmosphere with 5% CO_2_ at 37°C. All experiments were performed in serum-free medium. HiPerFect Transfection Reagent was obtained from Qiagen (Hilden, Germany). A First-Strand cDNA Synthesis kit and SYBR green real-time PCR Mastermix were supplied by Toyobo (Osaka, Japan). A Cell Counting Kit-8 (CCK-8) and Transwell product (Cat #3422) were purchased from Dojindo (Shanghai, China) and Corning (Tewksbury, MA, US), respectively. FITC Annexin V Apoptosis Detection Kit was obtained from BD Biosciences (Bedford, MA, US).

For western blot analysis, primary antibodies were purchased from Abcam (Cambridge, UK) (ASPP2, 1 : 5,000, rabbit polyclonal; *β*-catenin, 1 : 5,000, rabbit polyclonal; *α*-SMA, 1 : 4,000, rabbit polyclonal; fibronectin, 1 : 1,000, rabbit polyclonal; *β*-actin, 1 : 5,000, mouse monoclonal) and Cell Signaling Technology (ZEB1, 1 : 500, rabbit polyclonal; E-cadherin, 1 : 1,000, mouse monoclonal; zonula occludents-1 (ZO-1), 1 : 1,000, mouse monoclonal; *β*-tubulin, 1 : 500, rabbit polyclonal). Secondary antibodies conjugated with horseradish peroxidase against rabbit or mouse immunoglobulin obtained from Cell Signaling Technology (Danvers, MA, US) were used at a dilution of 1 : 1000. For immunochemistry analysis, anti-ASPP2 rabbit polyclonal antibody was used at a dilution of 1 : 500. Mouse monoclonal antibody specific for pan-cytokeratin (1 : 100) was obtained from Chemicon (Temecula, CA, US). Goat anti-rabbit tetramethylrhodamine isothiocyanate (TRITC) and goat anti-mouse fluorescein isothiocyanate- (FITC-) conjugated secondary antibodies were purchased from Molecular Probes (Invitrogen, Carlsbad, CA, US). All other reagents were supplied by Sigma-Aldrich (St. Louis, MO, US).

### 2.2. Human Tissue Sample Preparation

The Ethical Committee and Institutional Review Board of Peking University People's Hospital (Beijing, China) approved the human patient study protocol, which was conducted in accordance with the Declaration of Helsinki. Written informed consent was obtained from each study subject. The PVR specimens (*n* = 6) and the epiretinal membrane (ERM) specimens (*n* = 6) were dissected from patients with retinal detachment complicated by PVR and from patients with idiopathic epiretinal membrane during intraocular surgery (Table 1). Dr. Xiaoxin Li performed the vitrectomy surgeries in these patients.

All specimens were fixed in a test tube containing 4% phosphate-buffered saline (PBS, pH 7.4) and subsequently embedded in optimum cutting temperature compound (OCT) for immunohistochemistry. Thawed tissue sections were air dried, fixed in 4% paraformaldehyde (PFA) for 20 min, and blocked with 10% normal goat serum for 1 h at 37°C. Then, anti-ASPP2 together with anticytokeratin antibodies were applied to the tissue sections at 4°C overnight and incubated for 1 h at 37°C with 1 : 100 TRITC-conjugated goat anti-rabbit and FITC-conjugated goat anti-mouse antibodies. Following incubation, slides were washed with PBS and cell nuclei were stained with 4′,6′-diamino-2-phenylindole (DAPI). Images were acquired with a fluorescence microscope (Leica, Heidelberg, Germany). For each immunostaining, negative controls included the use of an isotype-matched monoclonal primary antibody, while the other procedures remained the same.

### 2.3. Small Interfering RNA and Transfection Assays

The ASPP2-specific (GenBank accession number NM_001031685) siRNA (ASPP2-siRNA: forward: 5′-AGG GAG TGT TTG AAT AAG C-3′; reverse: 5′-CAC CCA GAG AAC ATT TAT T-3′) was chemically synthesized by Qiagen. ARPE-19 cells were transfected with siRNA using HiPerFect Transfection Reagent according to the manufacturer's instructions as we previously described [27]. Briefly, siRNA was first suspended in siRNA suspension buffer and then aliquoted in the required amounts for each experiment. On the day of transfection, cells were seeded in plates at the recommended density. The siRNA was then gently introduced into the cells by mixing with the required amount of HiPerFect Transfection Reagent. Scrambled control-siRNA was used to control for any effects of the transfection reagent and siRNA. In our study, the final concentration of siRNA was 10 nM, and assays were performed 48 h after transfection unless otherwise stated.

### 2.4. Real-Time Quantitative RT-PCR

Total RNA was isolated with Trizol (Invitrogen, Carlsbad, CA, US) according to the manufacturer's protocol. Real-time quantitative PCR analysis was performed using IQ Supermix (Bio-Rad, Hercules, CA). The primers for ASPP2 were forward (5′-TTC GGG TCCAAGATG ATG CC-3′) and reverse (5′-CAA CTG GAC GTT CAG AGC CA-3′) and for glyceraldehyde phosphate dehydrogenase (GAPDH) were forward (5′-GAA GGT GAA GGT CGG AGT C-3′) and reverse (5′-GAA GAT GGT GAT GGG ATT TC-3′). The amplification and thermocycling procedures were conducted according to the manufacturer's instructions. GAPDH served as the reference gene for quantity control. Experiments were performed in triplicate and repeated at least three times.

### 2.5. Western Blot Analysis

Whole cell protein and cytoplasmic protein fractions were prepared with total protein extraction kit (Amsbio, Abingdon, UK) and NE-PER extraction reagent (Thermo Fisher Scientific, Rockford, US), respectively, according to the manufacturer's instructions. Then, protein concentrations were measured with a BCA Protein Assay Kit (Pierce, Rockford, IL, US). Equal amounts of protein were separated by 10% sodium dodecyl sulfate polyacrylamide gel and visualized with enhanced chemiluminescence detection reagents (Pierce). Each band density was quantified using Quantity One software (Bio-Rad, Richmond, CA, US) and normalized to *β*-actin or *β*-tubulin. All immunoblot analyses were repeated three times, and similar results were obtained.

### 2.6. Immunocytochemistry

For immunocytochemical staining, ARPE-19 cells grown on glass coverslips were washed in PBS, fixed in 4% PFA for 15 min, and blocked in 10% goat serum with 0.1% Triton X-100. Cells were then incubated with primary antibodies overnight at 4°C, followed by incubation with fluorescently labeled secondary antibodies for 1 h at room temperature in the dark. After three washes with PBS, cell nuclei were counterstained with DAPI. Images were visualized and acquired by a fluorescence microscope (Leica). Experiments were performed in triplicate and repeated three times.

### 2.7. Cell Apoptosis Assay

Cytotoxicity of siRNA transfection to ARPE-19 cells was determined by examining cell apoptosis using a FACS Caliber cytometer (Becton Dickinson, San Jose, CA, US) according to the manufacturer's instructions. In brief, ARPE-19 cells were plated at 1 × 10^6^ per well in 6-well plates. Cells with ASPP2-siRNA transfection and two control groups were used: normal control cells without transfection treatment (NC) and cells with scrambled control-siRNA transfection (SC). After 48 h incubation, cells were detached using ethylenediaminetetraacetic acid (EDTA), washed in ice-cold PBS, and treated with the FITC Annexin V Apoptosis Detection Kit.

### 2.8. Proliferation Assay

Cell proliferation was measured by a CCK-8 assay. ARPE-19 cells were treated as described above for 48 h and then detached by trypsin, counted, and allocated evenly to 96-well plates. From that time on, the CCK-8 assay was performed at 12, 24, 48, and 72 h according to the manufacturer's instructions and read by an ELISA microplate reader (Finstruments Multiskan Models 347; MTX Lab Systems, Inc., Vienna, VA).

### 2.9. Cell Migration

The ARPE-19 cell migration assay was performed using the Transwell assay. Briefly, 2 × 10^4^ cells in 200 *μ*L of serum-free medium were placed in the upper chamber of the Transwell. Next, DMEM with 10% FBS was placed in the bottom chamber at a final volume of 600 *μ*L. All migration assays were conducted for 6 h at 37°C. In the end, cells were fixed in 4% PFA and stained with DAPI for 15 min. The remaining cells were carefully removed using a cotton swab. The membrane was imaged, and cells from five random fields of view were counted. Each experiment was repeated three times.

### 2.10. Cytometric Bead Array

The concentrations of interleukin-1*β*, interleukin-6, interleukin-8, interleukin-10, interleukin-12p (IL-1*β*, IL-6, IL-8, IL-10, and IL-12p) and tumor necrosis factor-alpha (TNF-*α*) in ARPE-19 cell supernatants were measured with a cytometric bead array (CBA, number 552932; BD Bioscience, San Jose, CA, US) as previously described [28]. Briefly, cell supernatants were collected and centrifuged at 12,000 ×g at 4°C. Then, 50 *μ*L of the supernatant sample was used for each test and measured with flow cytometry (BD FACSCalibur; BD Bioscience). Quality control procedures were performed, and the concentration of each cytokine in one sample was calculated according to the protocol of the CBA kit.

### 2.11. PVR Model Induction and Treatment of siRNA

All experiments adhered to the ARVO statements for the Use of Animals in Ophthalmology and Vision Research and were approved by the Animal Care Use Committee of Peking University. All surgery was performed under sodium pentobarbital anesthesia, and all efforts were made to minimize suffering. Experimental PVR models were induced as previously reported with modifications [29]. In brief, forty-two specific pathogen-free male Brown Norway (BN) rats (200 ± 10 g, 6-7 weeks) were used in the present study. Rats were anesthetized, and pupils were dilated. Twenty-one rats were randomly assigned to receive intravitreal injection of ARPE-19 cells transfected with ASPP2-siRNA in the left eyes, and the other rats were treated with SC in the left eyes. PVR induction was performed by injecting 1 × 10^6^ ARPE-19 cells, transfected with chemically modified ASPP2-specific siRNA, in 10 *μ*L of balanced salt solution, into the vitreous cavity with a Hamilton syringe fitted with a 32-gauge microneedle. Rats of the SC group underwent intravitreal injections in a very similar way but had the ARPE-19 cells transfected with chemically modified scrambled control-siRNA. All injected eyes were ophthalmoscopically examined by two masked observers on days 1, 3, 7, 14, 21, and 28 after injection. Retinal photographs of each rat were taken using photography (Phoenix Micron IV Retinal Imaging Microscope, Pleasanton, CA, US). PVR was classified into four stages using the clinical criteria published by Behar-Cohen et al. (Table 2) [30].

### 2.12. Retinal Immunohistochemistry and Histology Examination

By the 14 d of the follow-up, animals were sacrificed by cervical dislocation, and retinal cryosections (10 *μ*m) were prepared as previously described [31]. For immunohistochemistry, primary antibodies (anti-ASPP2 and anti-cytokeratin antibody) were added to the retinal sections in blocking solution and incubated overnight at 4°C. Sections were washed and incubated with secondary antibodies: TRITC-conjugated goat anti-rabbit and FITC-conjugated goat anti-mouse antibodies. Fluorescent labeling was observed with a Leica microscope. For histology, retinal sections were stained with hematoxylin-eosin (H-E) and examined by a light microscope.

### 2.13. Statistical Analysis

Data were expressed as the mean ± standard deviation (SD), unless otherwise indicated. Statistical analysis was conducted using Prism 5 (GraphPad Software, Inc., San Diego, CA, US). Differences were evaluated using ANOVA followed by the Bonferroni* post hoc *test for multiple comparisons, or using Student's *t*-test for pairwise comparisons. The Wilcoxon rank sum test was performed for nonparametric ordinal data. *P* < 0.05 was considered to be statistically significant.

## 3. Results

### 3.1. Immunohistochemical Detection of ASPP2 in PVR Membranes

**Figure 1:**
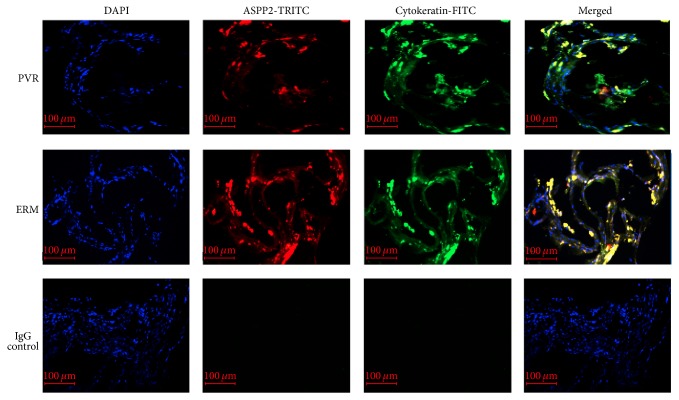
ASPP2 expression in PVR membranes. Fluorescence micrograph showing colocalization of ASPP2 (red) and cytokeratin (green) in both PVR membranes and ERM. Cell nuclei were stained with DAPI (blue). Isotype IgG was used for the negative control. Representative results obtained from samples of patients number 1 and number 10 are shown. PVR, proliferative vitreoretinopathy; ERM, epiretinal membranes; DAPI, 4′,6′-diamino-2-phenylindole. Scale bars, 100 *μ*m.

To investigate the expression of ASPP2 in PVR membranes, we stained sections with anti-ASPP2 and anti-cytokeratin antibodies. The positive staining demonstrated that ASPP2 was expressed in PVR membranes and coexisted with cytokeratin, which is a marker of RPE cells. Additionally, ASPP2 expression was less intense in PVR membranes compared to ERM (Figure 1).

### 3.2. Knockdown of ASPP2 in ARPE-19 Cells Promoted Cell Proliferation and Migration

**Figure 2:**
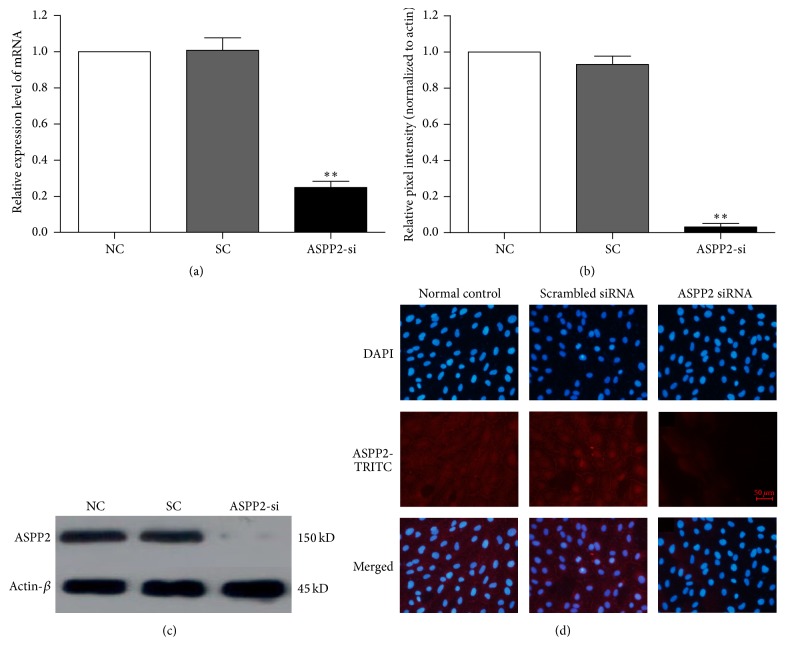
Knockdown of ASPP2 with siRNA efficiently inhibits ASPP2 expression. (a) ASPP2 mRNA expression was significantly downregulated in ARPE-19 cells 48 h after transfection, as measured by real-time RT-PCR. (b) The data of the relative ASPP2 protein in the NC, SC, and ASPP2-siRNA treatment group, normalized to *β*-actin. (c) Representative western blot showing the decreased protein expression of ASPP2 in ARPE-19 cells with ASPP2-siRNA transfection. (d) Cells stained with antibody against ASPP2 (red) and cell nuclei with DAPI were examined by fluorescence microscopy. The fluorescence intensity represents the protein expression of ASPP2 in ARPE-19 cells. Data are shown as the mean ± SD, *n* = 3. ^*∗∗*^ *P* < 0.01 versus NC or SC group. NC was set to 100%. NC, normal control cells; SC, scrambled control-siRNA-transfected cells; ASPP2-si, ASPP2-siRNA-transfected cells; DAPI, 4′,6′-diamino-2-phenylindole. Scale bars, 50 *μ*m.

ASPP2-siRNA was effectively transfected into ARPE-19 cells, and ASPP2 expression was significantly reduced at both the mRNA (*P* < 0.01, compared to SC group) (Figure 2(a)) and protein levels (*P* < 0.01, compared to SC group; Figures 2(b) and 2(c)). In contrast, there was no significant difference between the NC and SC groups (Figure 2, *P* > 0.05). Immunocytochemical imaging showed that ASPP2 was mainly expressed in the nucleus and the cytoplasm, as well as at the cell junction of ARPE-19 cells. Knockdown of ASPP2 was further confirmed in the immunocytochemistryassay (Figure 2(d)).

**Figure 3:**
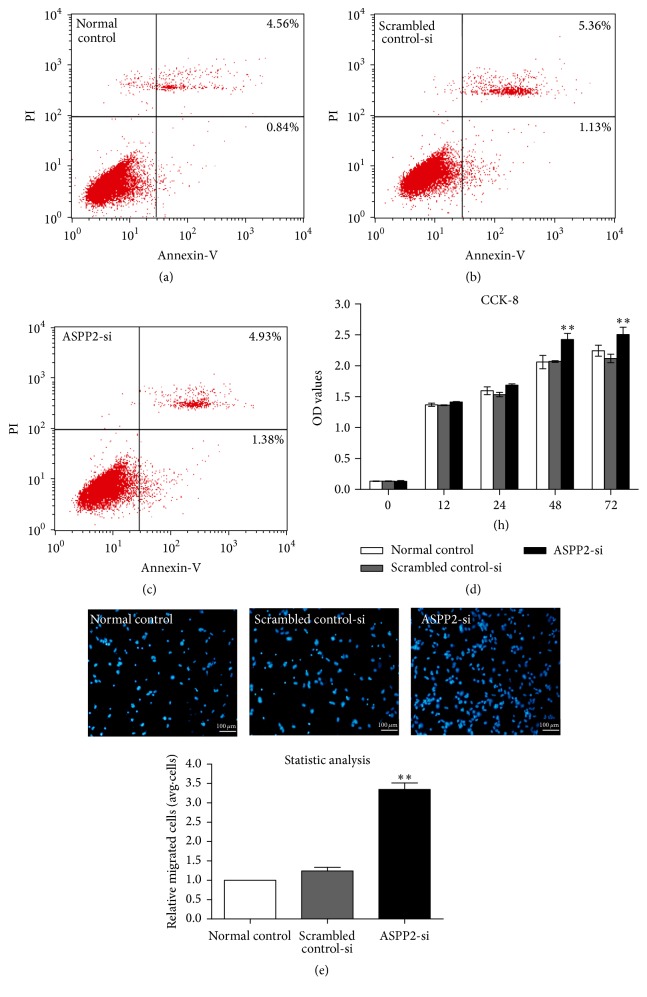
Cytotoxicity, proliferation, and migration induced by transfection of ARPE-19 cells. Normal control ARPE-19 cells without treatment and cells transfected with scrambled control-siRNA or ASPP2-siRNA were incubated for 48 h. Then, cells were stained with Annexin V and propidium iodide, followed by flow cytometry measurement. Rates of early apoptotic cells and late apoptotic cells are shown in the bottom right and top right quadrants, respectively (a, b, and c). Representative results of three independent experiments are shown. Cell proliferation was measured with a CCK-8 assay at 0, 12, 24, 48, and 72 h (d). Cell migratory activity was determined by Transwell assay 48 h after transfection (e). Statistical analysis based on the number of cells that had migrated through the filter of the chamber, showing that ASPP2 knockdown promoted cell migration significantly. Data are shown as the mean ± SD, *n* = 4 experiments. ^*∗∗*^ *P* < 0.01 versus normal control or Scrambled control-siRNA group.

To detect the cytotoxicity induced by siRNA transfection, we performed a cell apoptosis assay. There was no significant difference in cell apoptosis between the NC, SC, and ASPP2-siRNA treatment groups (Figures 3(a), 3(b), and 3(c), *P* > 0.05). In the CCK-8 assay, ASPP2-siRNA treatment increased proliferation of ARPE-19 cells significantly at 48 and 72 h, when compared with the NC and SC groups (Figure 3(d), *P* < 0.01).

Because enhanced migration is an important characteristic of EMT, we next examined the migration of ARPE-19 cells. The Transwell assay revealed that cellular migration activity increased approximately 2-fold in the ASPP2-siRNA treatment group compared with the NC and SC groups (Figure 3(e), *P* < 0.01). However, no significant difference was observed between the NC and SC groups for either cell proliferation or migration capacity.

### 3.3. ASPP2 Knockdown Facilitated EMT of ARPE-19 Cells

**Figure 4:**
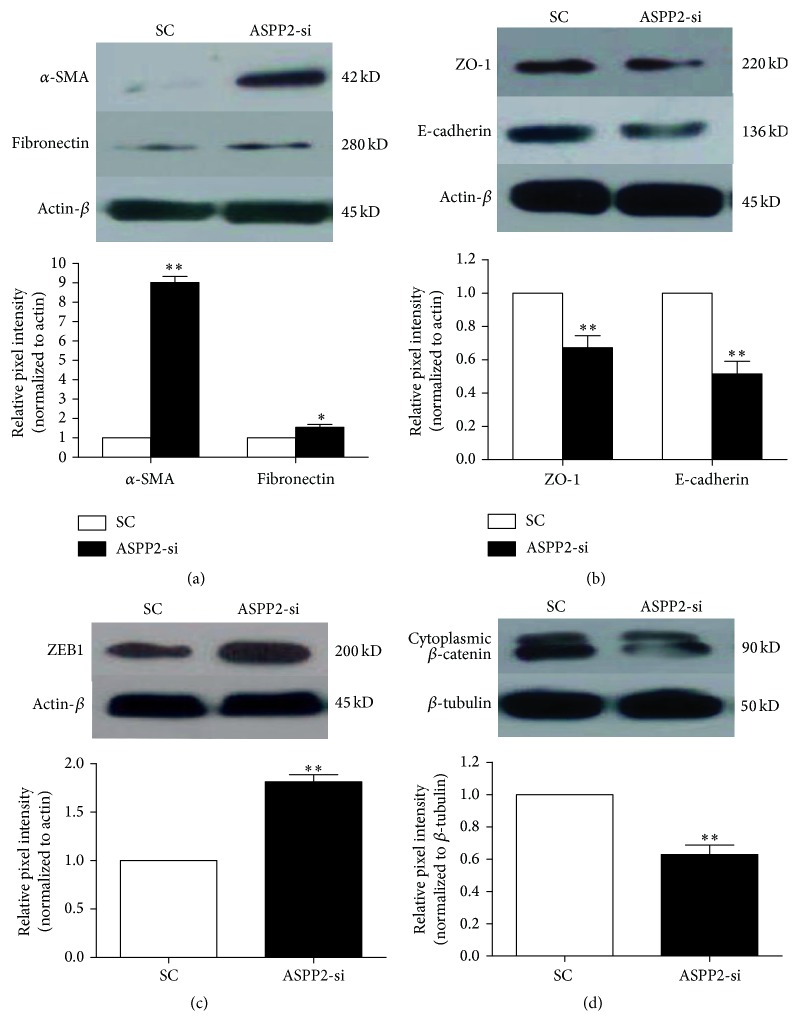
Effects of ASPP2 on the protein expression of epithelial and mesenchymal markers of ARPE-19 cells. Western blot images of expression of epithelial markers (ZO-1, E-cadherin, and *β*-catenin) were significantly decreased, while mesenchymal markers (*α*-SMA, fibronectin, and ZEB1) significantly increased in ARPE-19 cells 48 h after ASPP2-siRNA transfection, when compared to the scrambled control-siRNA transfection (a, b, c, and d). *β*-actin or *β*-tubulin was used as a loading control. Representative results obtained in three independent experiments are shown. Column diagrams and bars represent the ratio of the scanned immunoblots of mesenchymal markers and epithelial markers to *β*-actin or *β*-tubulin. Data are shown as the mean ± SD, *n* = 3 experiments. ^*∗*^ *P* < 0.05 versus SC group; ^*∗∗*^ *P* < 0.01 versus the SC group. SC was set to 100%. SC, scrambled control-siRNA-transfected cells; ASPP2-si, ASPP2-siRNA-transfected cells.

As for EMT, cells lose their epithelial characteristics and become more mesenchymal. Therefore, we investigated the role of ASPP2-knockdown in the induction of EMT of ARPE-19 cells by measuring epithelial markers and mesenchymal markers. Two days after ASPP2-knockdown of ARPE-19 cells, western blot assays were performed. Protein expression of epithelial markers (such as E-cadherin, ZO-1, and *β*-catenin) was downregulated (Figures 4(b) and 4(d), *P* < 0.01), whereas mesenchymal markers (such as *α*-SMA, fibronectin, and ZEB1) were upregulated significantly in the ASPP2-siRNA treatment group compared to the SC group (Figures 4(a) and 4(c), *P* < 0.05). Our data suggest that ARPE-19 cells with ASPP2-siRNA transfection acquired EMT phenotypes.

### 3.4. ASPP2 Knockdown Enhanced the Production of EMT-Related Cytokines

**Figure 5:**
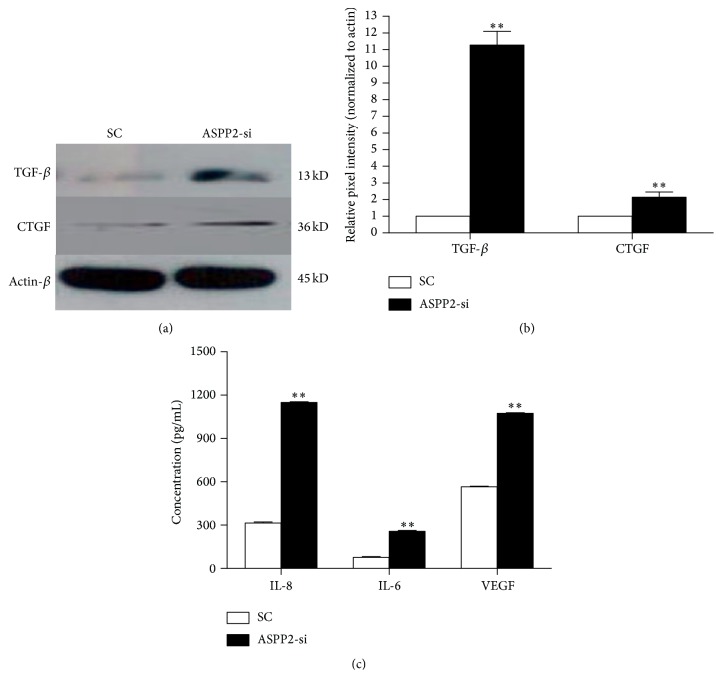
Effects of ASPP2 on the protein expression of EMT-related cytokines of ARPE-19 cells. After transfection with either scrambled control-siRNA or ASPP2-siRNA for 48 hours, protein levels of ARPE-19 cells were analyzed by western blot using antibodies against TGF-*β* and CTGF (a). *β*-actin was used as a loading control. Representative results obtained in three independent experiments are shown. Column diagrams and bars represent the ratio of the scanned immunoblots of TGF-*β* and CTGF to *β*-actin (b). SC was set to 100%. Meanwhile, concentrations of IL-8, IL-6, and VEGF in the culture supernatants of ARPE-19 cells were quantified with the CBA assay (c). Data are shown as the mean ± SD, *n* = 3 experiments. ^*∗∗*^ *P* < 0.01 versus SC group. SC, scrambled control-siRNA-transfected cells; ASPP2-si, ASPP2-siRNA-transfected cells.

Western blot assays were conducted to measure protein expression of TGF-*β* and CTGF, critical cytokines for EMT [32–34]. The CBA method was used to determine concentrations of secreted IL-1*β*, IL-6, IL-8, IL-10, IL-12p, VEGF, and TNF-*α*. Our results showed that production of TGF-*β* and CTGF in the ASPP2-siRNA treatment group was approximately 11-fold and 2-fold higher than in the SC group, respectively (Figures 5(a) and 5(b), *P* < 0.01). Additionally, the production of IL-8, IL-6, and VEGF was significantly higher in the ASPP2-siRNA treatment group than in the SC group (Figure 5(c), *P* < 0.01). However, there was no significant difference between these two groups in the secretion of the other cytokines examined, such as IL-1*β* and TNF-*α* (data not shown).

### 3.5. Effect of ASPP2 Knockdown on Induction of Experimental PVR

Our* in vitro* data suggested that ASPP2 plays an important role in proliferation and EMT of cultured ARPE-19 cells. Therefore, we employed an experimental rat model of PVR to further explore the role of ASPP2. Two rats were excluded because of cataracts 3 d after the injection. On day 7, vitreous haze and strands (stage 1) were observed in 10 rats (50%) of the ASPP2-siRNA treatment group, but no obvious changes were observed in the SC group. On day 14, eighteen rats (90%) of the ASPP2-siRNA treatment group developed to stage 2, whereas only 4 rats (20%) of the SC group showed stage 2. The difference was significant (*P* < 0.01). On day 28, thirteen rats (65%) in the ASPP2-siRNA treatment group displayed stage 3 PVR, with the remaining seven rats (35%) in stage 2. However, in the SC group, no rats progressed to stage 3 PVR, eight rats (40%) were in stage 2, and nine rats (45%) were in stage 1, while the three left did not show any obvious changes. The difference in severity of PVR between those two groups on day 28 was significant (*P* < 0.01) (see Table 3).

### 3.6. Retinal Morphological Changes after ASPP2-siRNA Administration

**Figure 6:**
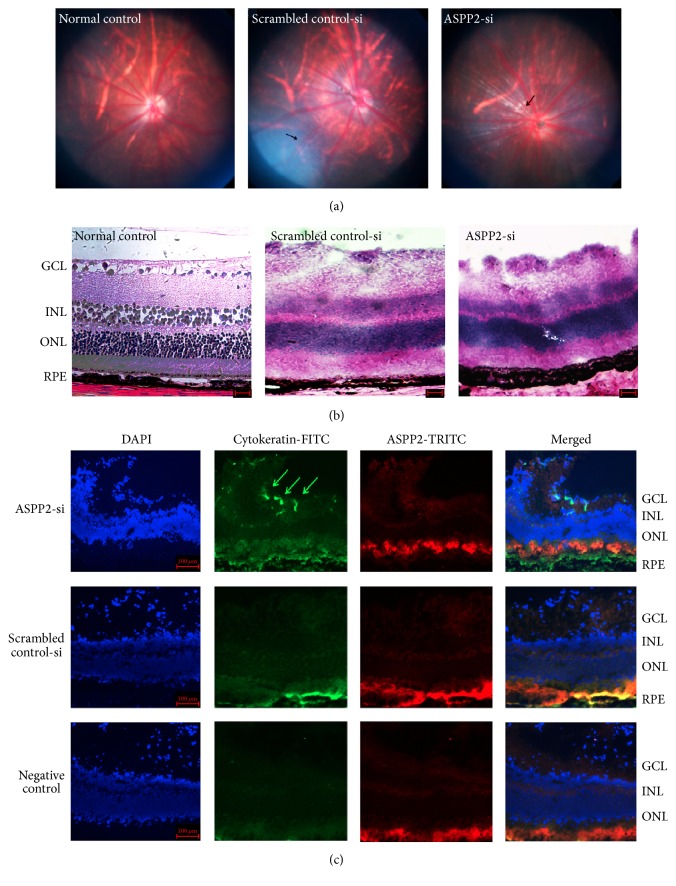
Effects of ASPP2 on retinal morphological changes of experimental PVR. Color fundus photographs of BN rats at 14-day follow-up (a). Fibrous proliferation was observed in both the scrambled control-siRNA treatment group and the ASPP2-siRNA treatment group, as indicated by the black arrows. Hematoxylin and eosin staining of retinal sections were performed at the 14-day follow-up (b). The swelling and proliferation of the ganglion cell layer in the scrambled control-siRNA and ASPP2-siRNA treatment group were apparent when compared to the normal control group. Scale bars, 50 *μ*m. Immunohistochemical staining of the retinal sections of the BN rats at the 14-day follow-up (c). Fluorescence micrograph showing that cytokeratin expression (green) existed in the ganglion cell layer of the ASPP2-siRNA treatment group (green arrows) but not in the scrambled control-siRNA treatment group or the negative control group. However, ASPP2 expression (red) was not colocalized with the cytokeratin expression in the ganglion cell layer in the ASPP2-siRNA treatment group, indicating the knockdown efficacy of ASPP2-siRNA treatment* in vivo*. Cell nuclei were stained with DAPI. Representative results obtained in at least three independent experiments are shown. DAPI, 4′,6′-diamino-2-phenylindole. Scale bars, 100 *μ*m.

To evaluate the proliferative effects of intravitreal injection of ARPE-19 transfected with ASPP2-siRNA, fundus photographs on day 14 were collected. More fibrosis changes were found in the ASPP2-siRNA treatment group when compared to the SC group (Figure 6(a)). Results from fundus studies were confirmed by histological examination of ocular specimens (Figure 6(b)). Ganglion cell layer swelling and proliferation were observed in both ASPP2-siRNA treatment and the SC group, but the ganglion cell layer swelling and proliferation were more pronounced in the former group (Figure 6(b)). To better characterize those changes, we used immunostaining for cytokeratin on retinal slides. Migration of pigmented epithelium cells into the ganglion cell layer was observed only in the ASPP2-siRNA treatment group at day 14, as evidenced by the positive staining of cytokeratin (Figure 6(c)). However, there was no observable cytokeratin expression in the ganglion cell layer for the SC group (Figure 6(c)).

## 4. Discussion

In the present study, we observed that ASPP2 was expressed in the PVR membranes and was significantly less intense compared to the ERM, indicating that ASPP2 probably plays a role in PVR pathogenesis. Furthermore, our* in vitro* experiments demonstrated that ASPP2-siRNA transfection promoted the proliferation and EMT of ARPE-19 cells, two important events in PVR development. Meanwhile, the* in vivo* studies showed that intravitreal injection of ARPE-19 cells transfected with ASPP2-siRNA accelerated the onset and progression of PVR in rat models, which was consistent with our* in vitro* studies. Taken together, our research shed new light on the molecular mechanism of PVR.

Although ASPP2 was regarded at first as apoptosis-related, mounting evidence has shown that reduced ASPP2 protein levels in many forms of cancer promoted cell migration or EMT and recently was shown to correlate with poor patient prognosis [22, 31, 35]. Wilson et al. proved that siRNA-based ASPP2 knockdown is safe and effective in promoting retinal ganglion cell survival after axonal injury [26]. Consistent with these studies, our results demonstrated the effectiveness and safety of ASPP2-siRNA transfection and the increased cell proliferation and migration after transfection.

EMT plays a key role in PVR development, but the exact mechanism of EMT in RPE cells is unknown. A study by Tamiya et al. strongly suggested that the loss of cell-cell adhesion was responsible for initiating EMT and the proliferation of RPE cells [36]. Cell adhesion molecules, including ZO-1 and E-cadherin, have been shown to play an essential role in maintaining epithelial differentiation and cell proliferation [37–40]. Wang et al. reported that ASPP2 knockdown sensitized HKe3 cells to oncogenic RAS-induced EMT by downregulation of epithelial markers and upregulation of mesenchymal markers [22]. In our study, we also found that protein levels of ZO-1 and E-cadherin were decreased, while those of *α*-SMA and fibronectin were increased by ARPE-19 cells with ASPP2-siRNA transfection. Additionally, our study showed the reduced expression of cytoplasmic *β*-catenin and the intense expression of ZEB1 during EMT of ARPE-19 cells, which was in accordance with the research of Park et al. [41].

Inflammatory or fibrosis-related cytokines such as TNF-*α*, IL-1*β*, CTGF, VEGF, and TGF-*β*, which are known to trigger EMT changes, were expressed in choroidal neovascular tissues and have been reportedly secreted by RPE cells [42–44]. The vitreous fluid of PVR patients has been found to contain various cytokines including TGF-*β* and IL-6 [45, 46], which contribute to the induction of EMT of RPE cells. TGF-*β* is a potent chemoattractant that plays a key role in transforming RPE cells into mesenchymal fibroblastic cells and in inducing type I collagen and extracellular matrix synthesis in RPE cells [47]. TNF-*α* is a marker of active inflammation, and genetic analysis has identified a single nucleotide polymorphism of the TNF locus that predisposes the eye to PVR [48]. CTGF and VEGF, which are important to stimulant fibrosis, are reportedly involved in the pathogenesis of PVR and played vital roles in EMT of RPE [33]. Furthermore, the strong correlation between these cytokine levels and the degree of fibrosis in vitreoretinal disorders suggests that they are important factors in ocular fibrosis and contraction of fibrovascular tissue. Moreover, the interleukins stimulate the proliferation of many types of cell and promote the synthesis of collagen during wound healing [49]. Park et al. demonstrated that the production of EMT-related cytokines, including TGF-*β*1, VEGF, IL-6, IL-8, MCP-1, and TNF-*α*, was approximately 2-fold to 5-fold higher during EMT of ARPE-19 cells [41]. However, our study only confirmed the increased production of TGF-*β*, CTGF, IL-8, IL-6, and VEGF in ARPE-19 cells transfected with ASPP2-siRNA. The discrepancy may be explained by the different methods for EMT induction, as Park et al. used Epstein-Barr virus infection while we employed ASPP2-siRNA transfection.

Because we have demonstrated that ASPP2 knockdown promoted proliferation and EMT of ARPE-19 cells, it is reasonable to propose that ASPP2 may play a critical role in PVR pathogenesis. Therefore, to test our hypothesis, we used an experimental rat model of PVR, which has been developed to evaluate vitreoretinal fibrosis [29, 30]. Our data suggest that intravitreal injection of ARPE-19 cells transfected with ASPP2-siRNA accelerated the onset and progression of PVR in rat models. The siRNA used in our study is a long-acting siRNA with improved stability by chemical modification, which has previously been reported by our laboratory [27].

There are some limitations in our study that should be noted. The ARPE-19 cells may not reflect EMT changes as accurately as primary cultured RPE cells, although the human RPE cell line is well acknowledged and widely used [8, 50, 51]. Besides, the PVR model established in this study is different from the human PVR. Nevertheless, since no perfect PVR model has been established yet, it is to justify using them for the investigation of PVR pathogenesis with proper orientation [5]. Furthermore, the sample size of PVR and ERM is slightly small, and it is not clear whether other cells such as glial cells have ASPP2 expression. Further studies are needed to confirm the role of ASPP2 in PVR pathogenesis.

In conclusion, our data demonstrate a novel and prominent role for ASPP2 in the pathogenesis of PVR, as indicated by ASPP2 knockdown, which not only promoted proliferation and EMT of ARPE-19 cells* in vitro* but also the onset and progression of experimental PVR* in vivo*, possibly via upregulating the expression of inflammatory and fibrosis cytokines. In the near future, we will focus on figuring out which exact cytokine is playing a key role in the ASPP2 knockdown event and hopefully develop novel strategies for the prevention and treatment of proliferative vitreoretinopathy.

## Figures and Tables

**Table 1:** Clinical characteristics for individual proliferative retinal membranes.

Patient number	Age (y)	Sex	Diagnosis	Duration
1	56	M	Idiopathic epiretinal membrane	3 mo
2	60	M	Idiopathic epiretinal membrane	5 wk
3	37	F	Idiopathic epiretinal membrane	2 mo
4	32	F	Idiopathic epiretinal membrane	1 wk
5	54	F	Idiopathic epiretinal membrane	6 mo
6	68	F	Idiopathic epiretinal membrane	4 mo
7	44	M	PVR epiretinal membrane	1 y
8	23	M	PVR epiretinal membrane	3 wk
9	30	M	PVR epiretinal membrane	2 mo
10	51	M	PVR epiretinal membrane	3 mo
11	37	F	PVR epiretinal membrane	5 mo
12	28	F	PVR epiretinal membrane	1 mo

Notes: PVR, proliferative vitreoretinopathy; M, male; F, female; mo, month; wk, week; y, year.

**Table 2:** Criteria for stages of proliferative vitreoretinopathy (adapted from [30]).

Stages	Characteristics
0	No proliferative response
1	Intravitreal proliferation
2	Preretinal membrane formation with retinal folds
3	White dense membrane covering the retina with retinal folds, localized retinal detachments, with or without localized posterior capsular cataract

**Table 3:** 

	D7			D14			D28		
	si (%)	sc (%)	*P*	si (%)	sc (%)	*P*	si (%)	sc (%)	*P*
Stage 0	10 (50%)	20 (100%)	<0.01	0	11 (55%)	<0.01	0	3 (15%)	<0.01
Stage 1	10 (50%)	0		2 (10%)	5 (25%)		0	9 (45%)	
Stage 2	0	0		18 (90%)	4 (20%)		7 (35%)	8 (40%)	
Stage 3	0	0		0	0		13 (65%)	0	
